# Supplementary material for: Synthesis and Assessment of Novel Sustainable Antioxidants with Different Polymer Systems
Source: Polymers (Basel). 2024 Feb 1;16(3):413. doi: 10.3390/polym16030413 (PMC10857301; doi:10.3390/polym16030413)
Supplement: Supplementary file 1 [file polymers-16-00413-s001.zip › polymers-2746053-supplementary.pdf]

SUPPORTING INFORMATION

Synthesis and assessment of novel sustainable antioxidants with different polymer systems.

Agathe Mouren, Eric Pollet, Luc Avérous*

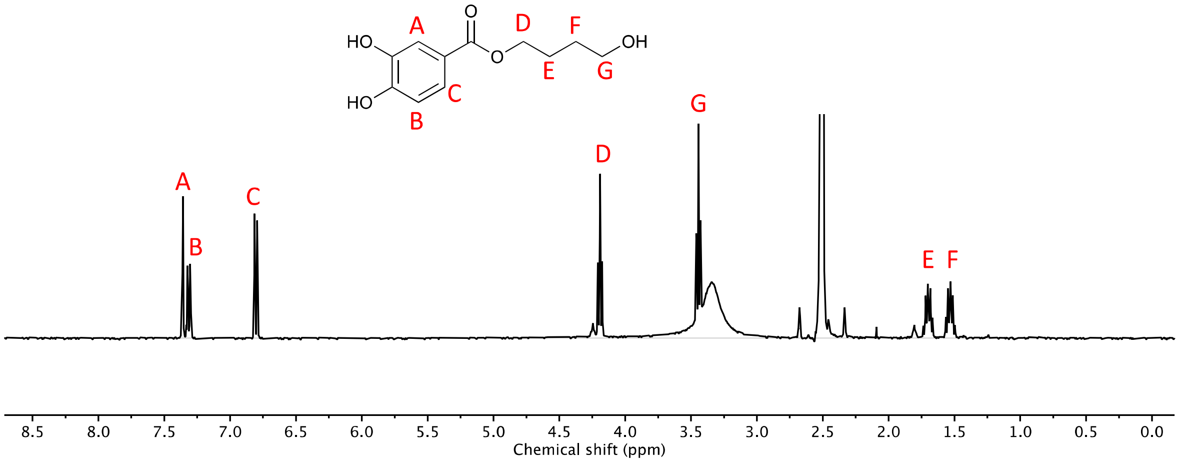

Figure S1. ***^{1}H NMR spectrum of BDO-3,4DHB in DMSO-d6.***

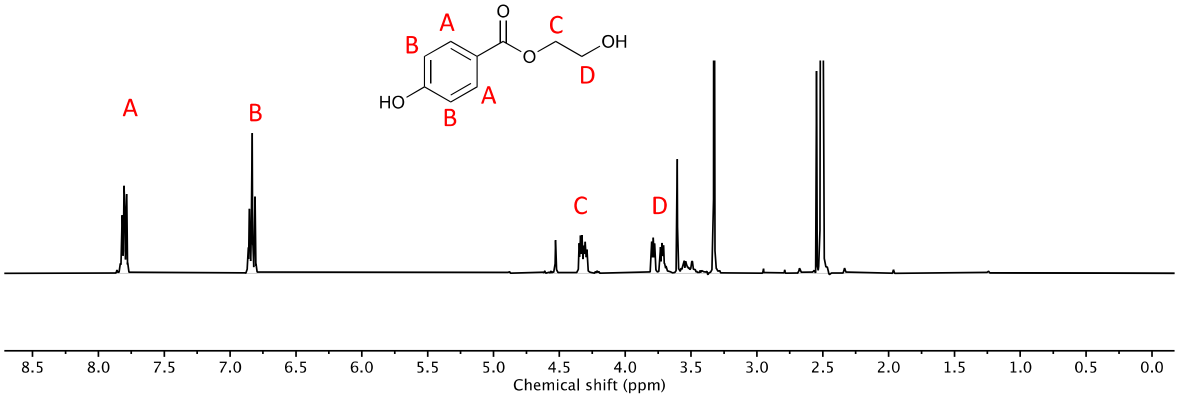

Figure S2. ***^{1}H NMR spectrum of EG-4HB in DMSO-d6.***

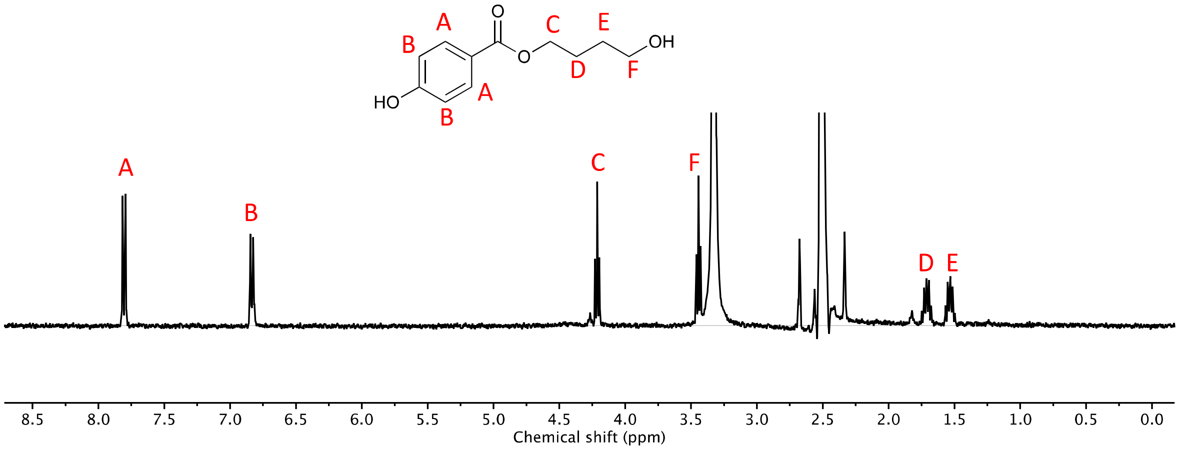

Figure S3.** ***^{1}H NMR spectrum of BDO-4HB in DMSO-d₆.

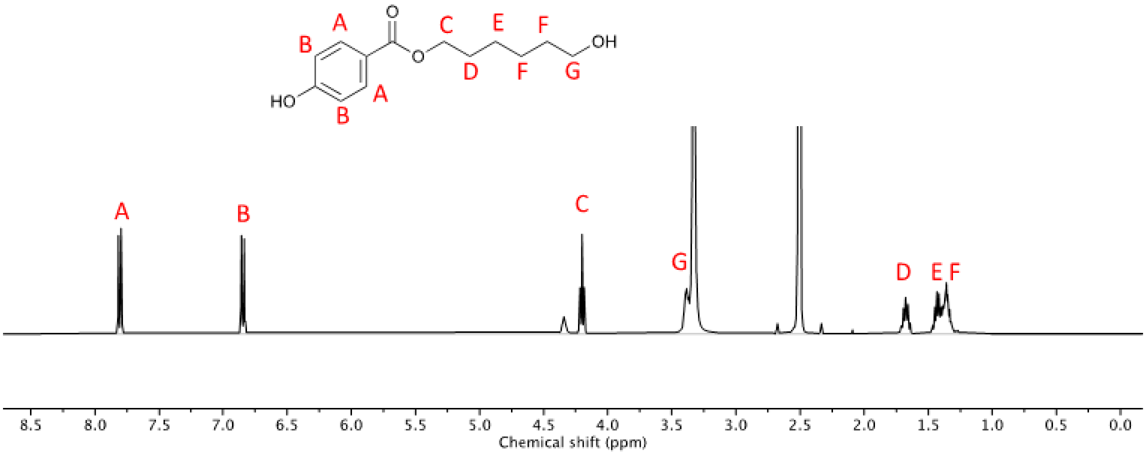

Figure S4.** ***^{1}H NMR spectrum of HDO-4HB in DMSO-d₆.

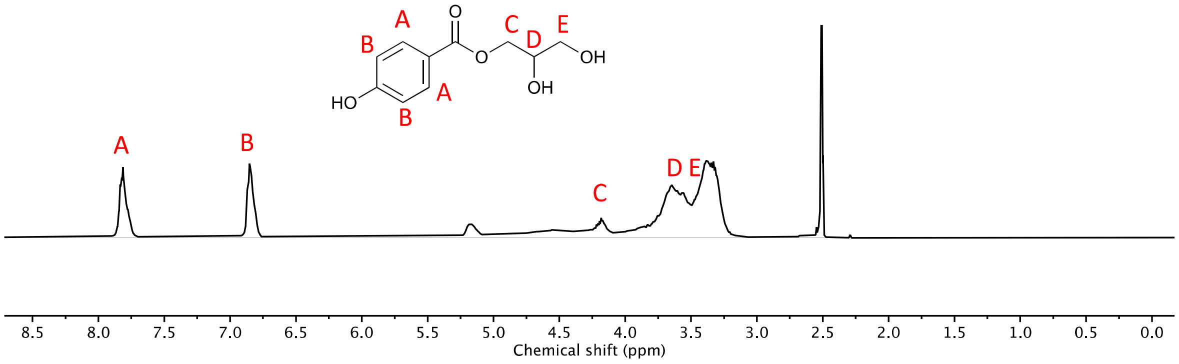

Figure S5.** ***^{1}H NMR spectrum of glycerol-4HB in DMSO-d₆.

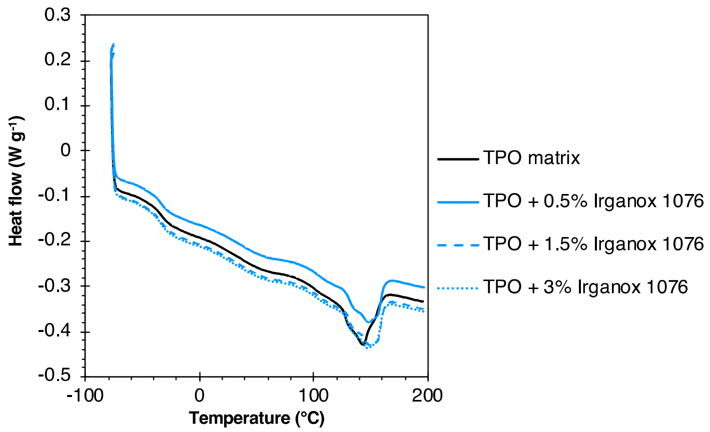

Figure S6. Evolution of DSC curves on the second heating of TPO formulations with different concentrations of Irganox 1076.

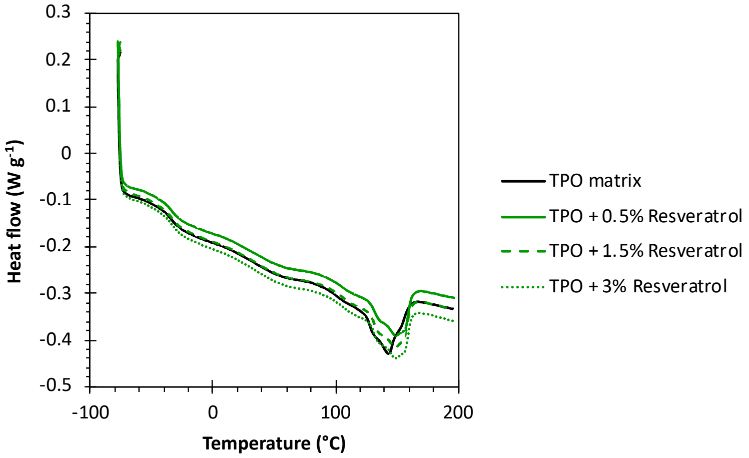

Figure S7. Evolution of DSC curves on the second heating of TPO formulations with different concentrations of resveratrol.

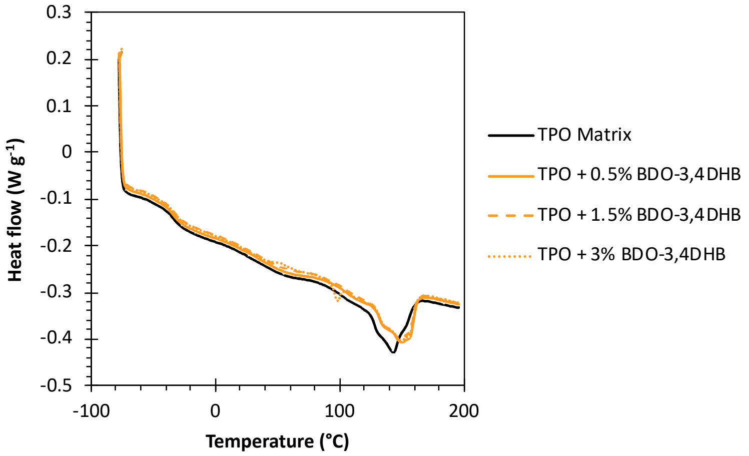

Figure S8. Evolution of DSC curves on the second heating of TPO formulations with different concentrations of BDO-3,4DHB.

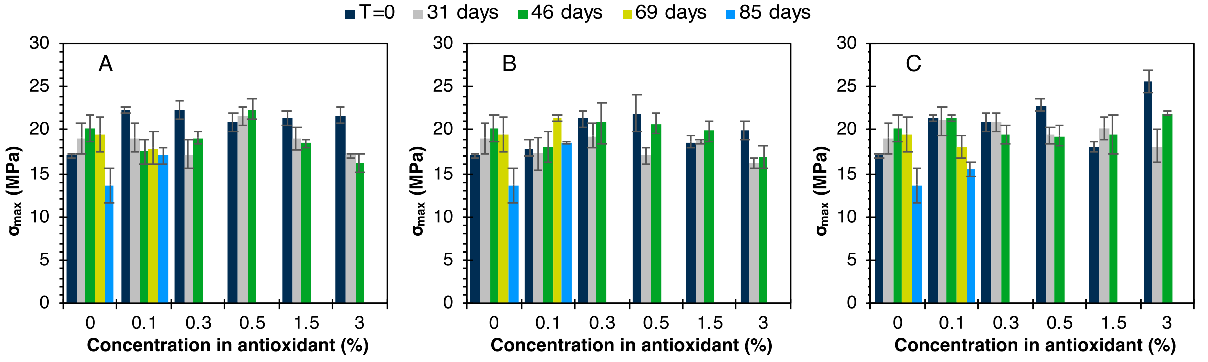

Figure S9. Stress at break at different aging times of TPO formulations with (A) Irganox 1076; (B) resveratrol, (C) BDO-3,4DHB.

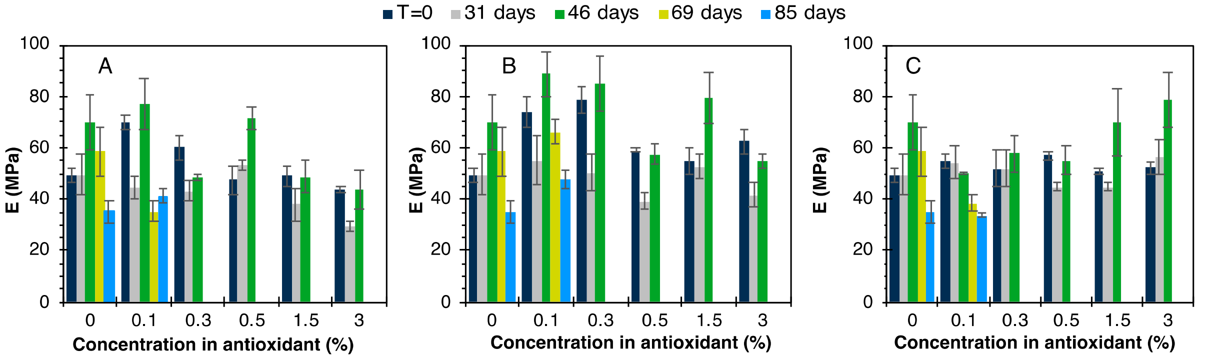

Figure S10. Young Modulus at different aging times of TPO formulations with (A) Irganox 1076; (B) resveratrol, (C) BDO-3,4DHB.

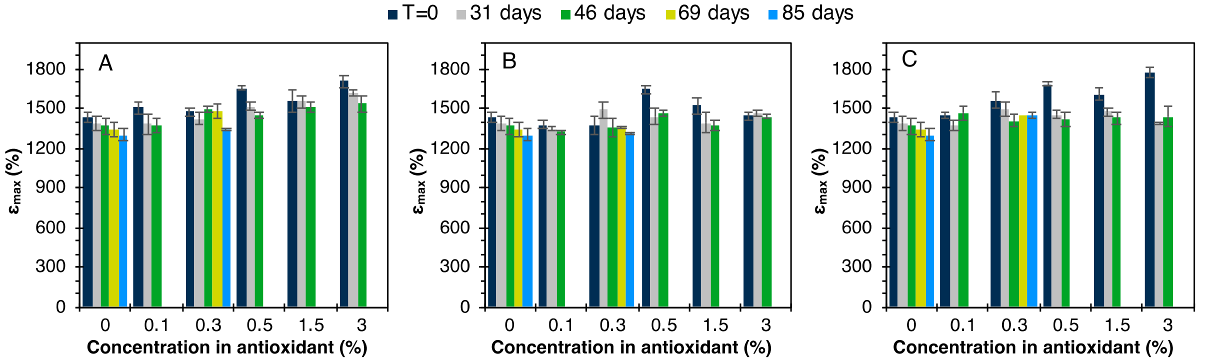

Figure S11. Elongation at break, at different aging times of TPO formulations with (A) Irganox 1076; (B) resveratrol, (C) BDO-3,4DHB.
